# 4-Amino­pyridinium 5-carb­oxy­penta­noate monohydrate

**DOI:** 10.1107/S1600536812027638

**Published:** 2012-06-23

**Authors:** S. Alfred Cecil Raj, A. Sinthiya, Babu Varghese

**Affiliations:** a Department of Physics, St. Josephs College (Autonomous), Tiruchirappalli 620 002, India; b Sophisticated Analytical Instruments Facility, Indian Institute of Technology Madras, Chennai 600 036, TamilNadu, India

## Abstract

In the title hydrated salt, C_5_H_7_N_2_^+^·C_6_H_9_O_4_^−^·H_2_O, the carb­oxy H atom is disordered over two positions with equal occupancy. In the crystal, O atoms of the 5-carb­oxy­penta­noate anion link the 4-amino­pyridinium cations and water mol­ecules into a three-dimensional network *via* N—H⋯O hydrogen bonds. The crystal structure is further consolidated by O—H⋯O hydrogen bonds involving the anion and the solvent water mol­ecule.

## Related literature

For the biological activity of 4-amino­pyridine, see: Judge & Bever (2006 ▶); Schwid *et al.* (1997 ▶); Strupp *et al.* (2004 ▶). For related structures, see: Anderson *et al.* (2005 ▶); Chao & Schempp (1977 ▶); Goswami & Ghosh (1997 ▶).
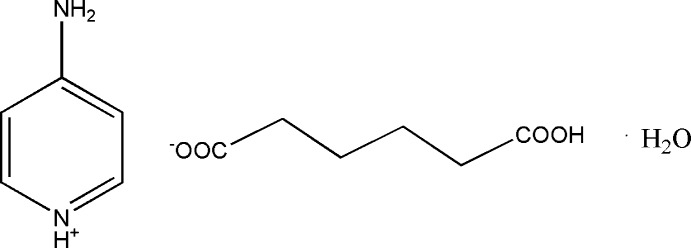


## Experimental

### 

#### Crystal data

C_5_H_7_N_2_^+^·C_6_H_9_O_4_^−^·H_2_O
*M* *_r_* = 258.27
Monoclinic, 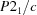

*a* = 11.9874 (6) Å
*b* = 5.1197 (2) Å
*c* = 21.5045 (9) Å
β = 96.498 (2)°
*V* = 1311.29 (10) Å^3^
*Z* = 4
Mo *K*α radiation
μ = 0.10 mm^−1^
*T* = 296 K
0.30 × 0.20 × 0.20 mm

#### Data collection

Bruker Kappa APEXII CCD diffractometer
Absorption correction: multi-scan (*SADABS*; Bruker, 2004 ▶) *T*_min_ = 0.970, *T*_max_ = 0.980
21231 measured reflections
3232 independent reflections
2737 reflections with *I* > 2σ(*I*)
*R*_int_ = 0.025

#### Refinement

*R*[*F* ^2^ > 2σ(*F* ^2^)] = 0.038
*wR*(*F* ^2^) = 0.110
*S* = 1.04
3232 reflections
184 parameters
6 restraints
H atoms treated by a mixture of independent and constrained refinement
Δρ_max_ = 0.24 e Å^−3^
Δρ_min_ = −0.17 e Å^−3^

### 

Data collection: *APEX2* (Bruker, 2004 ▶); cell refinement: *APEX2* and *SAINT* (Bruker, 2004 ▶); data reduction: *SAINT* and *XPREP* (Bruker, 2004 ▶); program(s) used to solve structure: *SIR92* (Altomare *et al.*, 1993 ▶); program(s) used to refine structure: *SHELXL97* (Sheldrick, 2008 ▶); molecular graphics: *ORTEP-3* (Farrugia, 1997 ▶) and *Mercury* (Macrae *et al.*, 2006 ▶); software used to prepare material for publication: *PLATON* (Spek, 2009 ▶).

## Supplementary Material

Crystal structure: contains datablock(s) global, I. DOI: 10.1107/S1600536812027638/bt5875sup1.cif

Structure factors: contains datablock(s) I. DOI: 10.1107/S1600536812027638/bt5875Isup2.hkl

Supplementary material file. DOI: 10.1107/S1600536812027638/bt5875Isup3.cml

Additional supplementary materials:  crystallographic information; 3D view; checkCIF report

## Figures and Tables

**Table 1:** Hydrogen-bond geometry (Å, °)

*D*—H⋯*A*	*D*—H	H⋯*A*	*D*⋯*A*	*D*—H⋯*A*
O2—H2*C*⋯O2^i^	0.82	1.63	2.4493 (18)	173
N1—H1*A*⋯O1^i^	0.88 (1)	1.93 (1)	2.7694 (15)	159 (2)
O4—H4*C*⋯O4^ii^	0.82	1.62	2.4320 (15)	168
N2—H2*A*⋯O3^ii^	0.88 (1)	1.96 (1)	2.8433 (13)	173 (1)
O1*S*—H1*S*⋯O1^iii^	0.85 (2)	1.98 (2)	2.8060 (16)	163 (2)
O1*S*—H2*S*⋯O1*S* ^iv^	0.85 (2)	1.97 (2)	2.8180 (11)	174 (2)
N2—H2*B*⋯O3^v^	0.88 (1)	2.07 (1)	2.9122 (13)	161 (1)

Symmetry codes: (i) 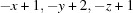; (ii) 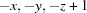; (iii) 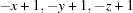; (iv) 
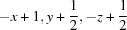
; (v) 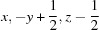.

**Fig. 1.**
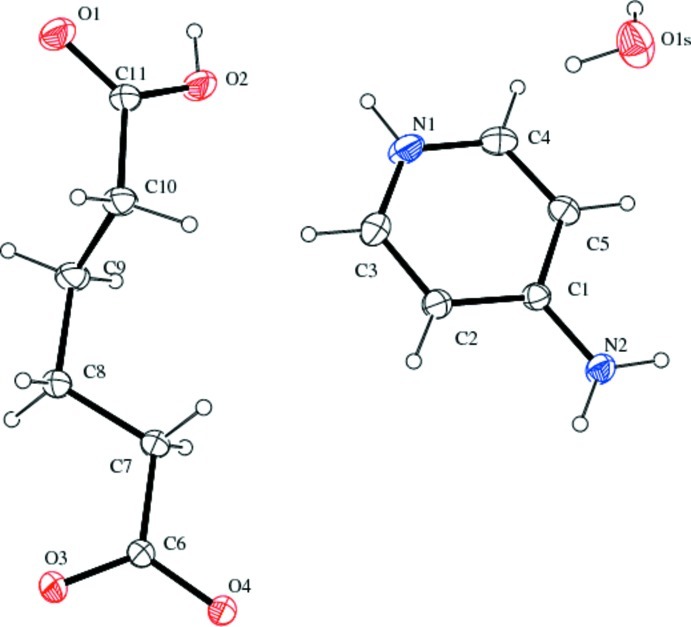
: The asymmetric unit of the title compound showing 30% probability displacement ellipsoids and the atomic numbering.

**Fig. 2.**
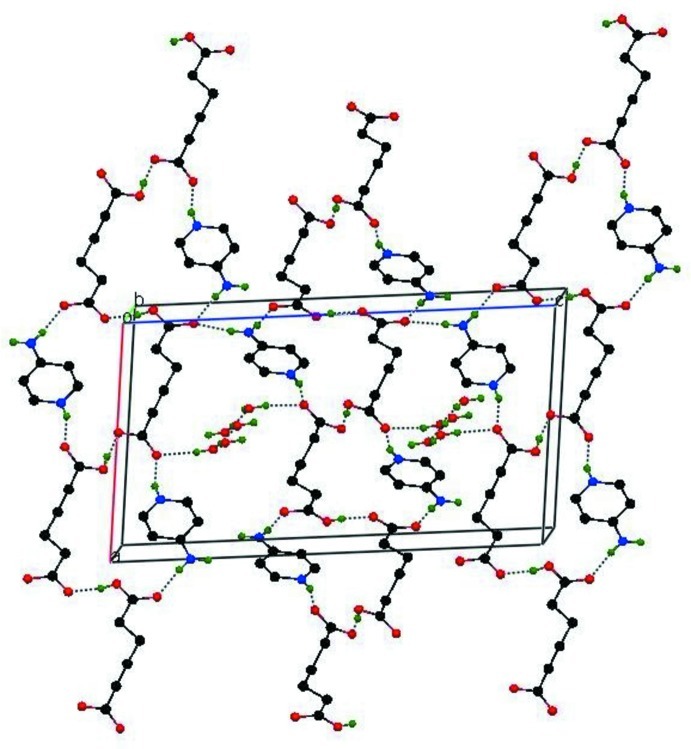
: The crystal packing of the title compound, viewed approximately down b axis. Hydrogen bonds are shown as dashed lines.

## supplementary crystallographic information

### Comment

4-Aminopyridine (Fampridine) is clinically used in the treatment of Lambert-Eaton myasthenic syndrome and multiple sclerosis. It prolongs action potentials by blocking potassium channels, thereby increases transmitter release at the neuromuscular junction (Judge & Bever, 2006; Schwid *et al.*, 1997; Strupp *et al.*, 2004). Hydrogen bonding plays a key role in the molecular recognition (Goswami & Ghosh, 1997).

The asymmetric unit of the title compound C_5_H_7_N_2_,C_6_H_9_O_4_,H_2_O contains one 4-aminopyridinium cation, one hydrogen adipate anion and one water molecule. In the hydrogen adipate anion the hydrogen atom of the COOH group is equally disordered (50:50) over two atomic sites. Figure 1 shows the asymmetric unit of the title compound C_5_H_7_N_2_,*C*_6_H_9_O_4_,H_2_O, showing 30% displacement ellipsoid probability and the atom numbering scheme. Cation link the oxygen ends of two adjacent carboxylate of anions. Bonding of the H atom to both pyridine ring N atom and amine group N atom of 4-aminopyridinium gives an ion to give the resonance structure.

The bond lengths and angles of 4-aminopyridinium cation agree with those previously reported (Chao & Schempp, 1977; Anderson *et al.*, 2005). A decrease in the C1–N2 bond length 1.3243 (17) Å is observed. Protonation of N1 of the 4-aminopyridinium results in widening of the C4–N1–C3, 120.41 (13)° which is 115.25 (3)° in the neutral 4-aminopyridinium molecule (Chao & Schempp, 1977; Anderson *et al.*, 2005).

In the molecular packing the title compound is mainly decided by N—H···O and O—H···O hydrogen bonds. The 4-aminopyridinium cations and hydrogen adipate anions are linked through two N—H···O and O—H···O hydrogen bonds (Table 1) forming an infinite molecular chain built from *R*_4_^4^(23) motif. The adjacent lattice water molecules in the crystal is linked through O1S—H2S···O1 hydrogen bond forming an infinite water chain extending along the [0 1 0] direction and the water chains connects the adjacent anionic-cationic chain building up a three dimensional network thus stabilizing the crystalline solid. The hydrogen bonded network is shown in Figure 2

### Experimental

All the reagents used for the preparation of sample are analytical grade and the solutions are prepared using pure deionized water. Solutions of 4 aminopyridine and adipic acid in water (20 ml) each are mixed in molar ratio of one isto two. The solution was uniformly stirred for 30 min and heated at 303 K for 2 h. The resulting solution was allowed to cool slowly to room temperature. Colorless crystals were obtained by slow evaporation after a period of two weeks.

### Refinement

The hydrogen atom of the carboxyl group, which is disordered over two sites with equal occupancy, was located in a difference electron density map and allowed to ride on the parent O atom with d(O–H) = 0.82 Å and *U*_iso_(H) = 1.5 *U*_eq_(O). The water H atoms and the H atoms bonded to N atoms were isotropically refined with distance restraints of d(O–H)=0.86 (2) Å and d(N–H)=0.88 (1) Å, respectively. The H···H distance in the water molecule was restrained to 1.36 (4)Å. The carbon H atoms were positioned geometrically and refined using a riding model with C–H = 0.93–0.97 Å and with *U*_iso_(H) = 1.2*U*_eq_(C) or 1.5*U*_eq_(C) for methyl groups.

### Crystal data

**Table:**

C_5_H_7_N_2_^+^·C_6_H_9_O_4_^−^·H_2_O	*F*(000) = 552
*M**_r_* = 258.27	*D*_x_ = 1.308 Mg m^−^^3^
Monoclinic, *P*2_1_/*c*	Mo *K*α radiation, λ = 0.71073 Å
Hall symbol: -P 2ybc	Cell parameters from 9976 reflections
*a* = 11.9874 (6) Å	θ = 4.8–56.6°
*b* = 5.1197 (2) Å	µ = 0.10 mm^−^^1^
*c* = 21.5045 (9) Å	*T* = 296 K
β = 96.498 (2)°	Block, colourless
*V* = 1311.29 (10) Å^3^	0.30 × 0.20 × 0.20 mm
*Z* = 4	

### Data collection

**Table:**

Bruker Kappa APEXII CCD diffractometer	3232 independent reflections
Radiation source: fine-focus sealed tube	2737 reflections with *I* > 2σ(*I*)
Graphite monochromator	*R*_int_ = 0.025
ω and φ scan	θ_max_ = 28.4°, θ_min_ = 3.4°
Absorption correction: multi-scan (*SADABS*; Bruker, 2004)	*h* = −15→15
*T*_min_ = 0.970, *T*_max_ = 0.980	*k* = −6→6
21231 measured reflections	*l* = −28→28

### Refinement

**Table:**

Refinement on *F*^2^	Secondary atom site location: difference Fourier map
Least-squares matrix: full	Hydrogen site location: inferred from neighbouring sites
*R*[*F*^2^ > 2σ(*F*^2^)] = 0.038	H atoms treated by a mixture of independent and constrained refinement
*wR*(*F*^2^) = 0.110	*w* = 1/[σ^2^(*F*_o_^2^) + (0.0506*P*)^2^ + 0.3091*P*] where *P* = (*F*_o_^2^ + 2*F*_c_^2^)/3
*S* = 1.04	(Δ/σ)_max_ = 0.001
3232 reflections	Δρ_max_ = 0.24 e Å^−^^3^
184 parameters	Δρ_min_ = −0.17 e Å^−^^3^
6 restraints	Extinction correction: *SHELXL97* (Sheldrick, 2008), Fc^*^=kFc[1+0.001xFc^2^λ^3^/sin(2θ)]^-1/4^
Primary atom site location: structure-invariant direct methods	Extinction coefficient: 0.038 (3)

### Special details

**Table:**

Geometry. All e.s.d.'s (except the e.s.d. in the dihedral angle between two l.s. planes) are estimated using the full covariance matrix. The cell e.s.d.'s are taken into account individually in the estimation of e.s.d.'s in distances, angles and torsion angles; correlations between e.s.d.'s in cell parameters are only used when they are defined by crystal symmetry. An approximate (isotropic) treatment of cell e.s.d.'s is used for estimating e.s.d.'s involving l.s. planes.
Refinement. Refinement of *F*^2^ against ALL reflections. The weighted *R*-factor *wR* and goodness of fit *S* are based on *F*^2^, conventional *R*-factors *R* are based on *F*, with *F* set to zero for negative *F*^2^. The threshold expression of *F*^2^ > σ(*F*^2^) is used only for calculating *R*-factors(gt) *etc*. and is not relevant to the choice of reflections for refinement. *R*-factors based on *F*^2^ are statistically about twice as large as those based on *F*, and *R*- factors based on ALL data will be even larger.

### Fractional atomic coordinates and isotropic or equivalent isotropic displacement parameters (Å^2^)

**Table:**

	*x*	*y*	*z*	*U*_iso_*/*U*_eq_	Occ. (<1)
C1	0.15377 (9)	0.4793 (2)	0.31363 (5)	0.0355 (2)	
C2	0.17198 (10)	0.4887 (3)	0.37922 (5)	0.0471 (3)	
H2	0.1338	0.3748	0.4031	0.057*	
C3	0.24500 (12)	0.6634 (3)	0.40748 (6)	0.0583 (4)	
H3	0.2567	0.6687	0.4510	0.070*	
C4	0.28575 (11)	0.8266 (3)	0.31185 (7)	0.0540 (3)	
H4	0.3251	0.9439	0.2895	0.065*	
C5	0.21437 (10)	0.6572 (3)	0.28057 (6)	0.0456 (3)	
H5	0.2050	0.6576	0.2370	0.055*	
C6	0.05042 (9)	0.0846 (2)	0.59628 (5)	0.0365 (2)	
C7	0.12933 (10)	0.2870 (2)	0.57471 (5)	0.0399 (3)	
H7A	0.1793	0.2027	0.5485	0.048*	
H7B	0.0857	0.4158	0.5493	0.048*	
C8	0.19886 (11)	0.4250 (3)	0.62766 (6)	0.0484 (3)	
H8A	0.2410	0.2958	0.6537	0.058*	
H8B	0.1489	0.5131	0.6533	0.058*	
C9	0.28055 (11)	0.6242 (3)	0.60591 (6)	0.0497 (3)	
H9A	0.2408	0.7332	0.5738	0.060*	
H9B	0.3070	0.7360	0.6409	0.060*	
C10	0.38119 (10)	0.5025 (2)	0.58006 (6)	0.0451 (3)	
H10A	0.4197	0.3861	0.6110	0.054*	
H10B	0.3563	0.4010	0.5430	0.054*	
C11	0.46060 (10)	0.7138 (2)	0.56373 (6)	0.0428 (3)	
N1	0.30125 (10)	0.8299 (3)	0.37434 (6)	0.0569 (3)	
N2	0.08259 (9)	0.3108 (2)	0.28417 (5)	0.0453 (3)	
O1	0.54037 (8)	0.7786 (2)	0.60138 (4)	0.0611 (3)	
O2	0.43660 (8)	0.8222 (2)	0.51080 (4)	0.0612 (3)	
H2C	0.4821	0.9385	0.5065	0.092*	0.50
O3	0.04538 (8)	0.04862 (19)	0.65223 (3)	0.0499 (2)	
O4	−0.00945 (8)	−0.04767 (19)	0.55455 (4)	0.0529 (3)	
H4C	0.0036	0.0002	0.5197	0.079*	0.50
O1S	0.46992 (12)	0.2870 (2)	0.26985 (6)	0.0725 (3)	
H1S	0.4605 (19)	0.299 (4)	0.3083 (8)	0.097 (7)*	
H2S	0.4831 (18)	0.439 (3)	0.2567 (9)	0.086 (6)*	
H2A	0.0462 (12)	0.203 (3)	0.3067 (6)	0.053 (4)*	
H2B	0.0745 (12)	0.313 (3)	0.2430 (4)	0.053 (4)*	
H1A	0.3493 (14)	0.946 (3)	0.3920 (9)	0.086 (6)*	

### Atomic displacement parameters (Å^2^)

**Table:**

	*U*^11^	*U*^22^	*U*^33^	*U*^12^	*U*^13^	*U*^23^
C1	0.0339 (5)	0.0370 (5)	0.0351 (5)	−0.0015 (4)	0.0027 (4)	0.0036 (4)
C2	0.0455 (6)	0.0604 (8)	0.0347 (5)	−0.0141 (6)	0.0012 (4)	0.0058 (5)
C3	0.0531 (7)	0.0773 (10)	0.0425 (6)	−0.0154 (7)	−0.0037 (5)	−0.0034 (6)
C4	0.0480 (7)	0.0489 (7)	0.0664 (8)	−0.0129 (6)	0.0120 (6)	0.0056 (6)
C5	0.0490 (6)	0.0471 (7)	0.0417 (6)	−0.0078 (5)	0.0100 (5)	0.0066 (5)
C6	0.0401 (5)	0.0407 (6)	0.0288 (5)	−0.0098 (4)	0.0043 (4)	0.0018 (4)
C7	0.0452 (6)	0.0423 (6)	0.0328 (5)	−0.0134 (5)	0.0065 (4)	0.0032 (4)
C8	0.0515 (7)	0.0563 (7)	0.0397 (6)	−0.0230 (6)	0.0153 (5)	−0.0117 (5)
C9	0.0516 (7)	0.0442 (7)	0.0563 (7)	−0.0187 (5)	0.0188 (5)	−0.0135 (6)
C10	0.0440 (6)	0.0410 (6)	0.0514 (6)	−0.0128 (5)	0.0099 (5)	−0.0018 (5)
C11	0.0400 (6)	0.0465 (6)	0.0432 (6)	−0.0132 (5)	0.0102 (5)	−0.0047 (5)
N1	0.0461 (6)	0.0573 (7)	0.0655 (7)	−0.0173 (5)	−0.0017 (5)	−0.0092 (6)
N2	0.0523 (6)	0.0483 (6)	0.0341 (5)	−0.0157 (5)	0.0001 (4)	0.0034 (4)
O1	0.0579 (6)	0.0719 (7)	0.0512 (5)	−0.0319 (5)	−0.0036 (4)	0.0079 (5)
O2	0.0593 (6)	0.0768 (7)	0.0457 (5)	−0.0360 (5)	−0.0023 (4)	0.0098 (5)
O3	0.0613 (5)	0.0607 (6)	0.0280 (4)	−0.0249 (4)	0.0064 (3)	0.0027 (4)
O4	0.0644 (6)	0.0639 (6)	0.0303 (4)	−0.0343 (5)	0.0050 (4)	−0.0007 (4)
O1S	0.1089 (10)	0.0509 (6)	0.0616 (7)	−0.0080 (6)	0.0267 (7)	−0.0044 (5)

### Geometric parameters (Å, º)

**Table:**

C1—N2	1.3234 (15)	C8—H8A	0.9700
C1—C2	1.4036 (15)	C8—H8B	0.9700
C1—C5	1.4070 (15)	C9—C10	1.5179 (18)
C2—C3	1.3475 (19)	C9—H9A	0.9700
C2—H2	0.9300	C9—H9B	0.9700
C3—N1	1.3407 (19)	C10—C11	1.5085 (16)
C3—H3	0.9300	C10—H10A	0.9700
C4—N1	1.3357 (19)	C10—H10B	0.9700
C4—C5	1.3440 (19)	C11—O1	1.2266 (15)
C4—H4	0.9300	C11—O2	1.2697 (16)
C5—H5	0.9300	N1—H1A	0.882 (9)
C6—O3	1.2255 (12)	N2—H2A	0.883 (9)
C6—O4	1.2778 (13)	N2—H2B	0.879 (9)
C6—C7	1.5110 (14)	O2—H2C	0.8200
C7—C8	1.5084 (16)	O4—H4C	0.8200
C7—H7A	0.9700	O1S—H1S	0.850 (15)
C7—H7B	0.9700	O1S—H2S	0.850 (15)
C8—C9	1.5236 (16)		
			
N2—C1—C2	121.45 (10)	C7—C8—H8B	108.8
N2—C1—C5	121.47 (10)	C9—C8—H8B	108.8
C2—C1—C5	117.08 (10)	H8A—C8—H8B	107.7
C3—C2—C1	119.67 (11)	C10—C9—C8	113.76 (11)
C3—C2—H2	120.2	C10—C9—H9A	108.8
C1—C2—H2	120.2	C8—C9—H9A	108.8
N1—C3—C2	121.50 (12)	C10—C9—H9B	108.8
N1—C3—H3	119.3	C8—C9—H9B	108.8
C2—C3—H3	119.3	H9A—C9—H9B	107.7
N1—C4—C5	121.32 (12)	C11—C10—C9	109.86 (10)
N1—C4—H4	119.3	C11—C10—H10A	109.7
C5—C4—H4	119.3	C9—C10—H10A	109.7
C4—C5—C1	120.05 (12)	C11—C10—H10B	109.7
C4—C5—H5	120.0	C9—C10—H10B	109.7
C1—C5—H5	120.0	H10A—C10—H10B	108.2
O3—C6—O4	121.58 (10)	O1—C11—O2	123.65 (11)
O3—C6—C7	120.43 (10)	O1—C11—C10	120.38 (11)
O4—C6—C7	117.98 (9)	O2—C11—C10	115.92 (10)
C8—C7—C6	113.65 (9)	C4—N1—C3	120.38 (11)
C8—C7—H7A	108.8	C4—N1—H1A	116.8 (13)
C6—C7—H7A	108.8	C3—N1—H1A	122.8 (13)
C8—C7—H7B	108.8	C1—N2—H2A	118.6 (10)
C6—C7—H7B	108.8	C1—N2—H2B	117.6 (10)
H7A—C7—H7B	107.7	H2A—N2—H2B	123.8 (14)
C7—C8—C9	113.65 (10)	C11—O2—H2C	109.5
C7—C8—H8A	108.8	C6—O4—H4C	109.5
C9—C8—H8A	108.8	H1S—O1S—H2S	108 (2)
			
N2—C1—C2—C3	−179.88 (13)	C6—C7—C8—C9	178.54 (11)
C5—C1—C2—C3	0.02 (19)	C7—C8—C9—C10	−73.66 (16)
C1—C2—C3—N1	0.0 (2)	C8—C9—C10—C11	−176.35 (11)
N1—C4—C5—C1	0.3 (2)	C9—C10—C11—O1	94.81 (15)
N2—C1—C5—C4	179.76 (13)	C9—C10—C11—O2	−82.64 (15)
C2—C1—C5—C4	−0.14 (18)	C5—C4—N1—C3	−0.3 (2)
O3—C6—C7—C8	1.47 (17)	C2—C3—N1—C4	0.2 (2)
O4—C6—C7—C8	−177.95 (12)		

### Hydrogen-bond geometry (Å, º)

**Table:**

*D*—H···*A*	*D*—H	H···*A*	*D*···*A*	*D*—H···*A*
O2—H2*C*···O2^i^	0.82	1.63	2.4493 (18)	173
N1—H1*A*···O1^i^	0.88 (1)	1.93 (1)	2.7694 (15)	159 (2)
O4—H4*C*···O4^ii^	0.82	1.62	2.4320 (15)	168
N2—H2*A*···O3^ii^	0.88 (1)	1.96 (1)	2.8433 (13)	173 (1)
O1*S*—H1*S*···O1^iii^	0.85 (2)	1.98 (2)	2.8060 (16)	163 (2)
O1*S*—H2*S*···O1*S*^iv^	0.85 (2)	1.97 (2)	2.8180 (11)	174 (2)
N2—H2*B*···O3^v^	0.88 (1)	2.07 (1)	2.9122 (13)	161 (1)

Symmetry codes: (i) −*x*+1, −*y*+2, −*z*+1; (ii) −*x*, −*y*, −*z*+1; (iii) −*x*+1, −*y*+1, −*z*+1; (iv) −*x*+1, *y*+1/2, −*z*+1/2; (v) *x*, −*y*+1/2, *z*−1/2.
